# Publisher Correction: Pushing the thinness limit of silver films for flexible optoelectronic devices via ion-beam thinning-back process

**DOI:** 10.1038/s41467-024-47014-z

**Published:** 2024-03-22

**Authors:** Dongxu Ma, Ming Ji, Hongbo Yi, Qingyu Wang, Fu Fan, Bo Feng, Mengjie Zheng, Yiqin Chen, Huigao Duan

**Affiliations:** 1 College of Mechanical and Vehicle Engineering, Hunan University, Changsha, Hunan Province China; 2 IBD Technology Co., Ltd., Zhongshan, Guangdong Province China; 3 Greater Bay Area Institute for Innovation, Hunan University, Guangzhou, Guangdong Province China; 4 Jihua Laboratory, Foshan, Guangdong Province China

**Keywords:** Synthesis and processing, Surfaces, interfaces and thin films, Synthesis and processing, Electronic devices

Correction to: *Nature Communications* 10.1038/s41467-024-46467-6, published online 13 March 2024

In this article the wrong figure appeared as Fig. 1; the figure should have appeared as shown below.
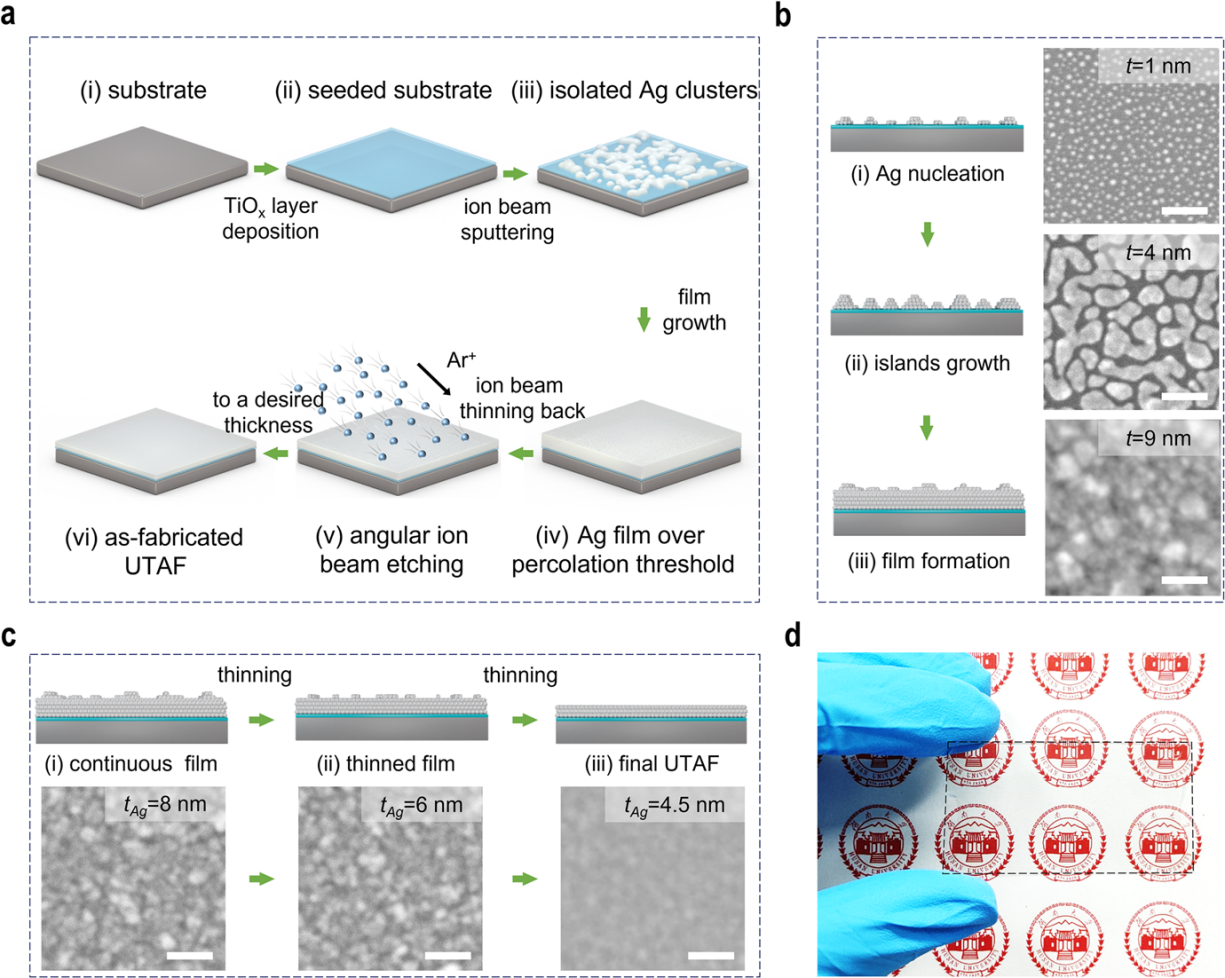


The original article has been corrected.

