# Supplementary material for: Pushing the thinness limit of silver films for flexible optoelectronic devices via ion-beam thinning-back process
Source: Nat Commun. 2024 Mar 13;15:2248. doi: 10.1038/s41467-024-46467-6 (PMC10933474; doi:10.1038/s41467-024-46467-6)
Supplement: Supplementary file 1 — Supplementary Information [file 41467_2024_46467_MOESM1_ESM.pdf]

Supplementary Information for

Pushing the thinness limit of silver films for flexible optoelectronic devices via ion-beam thinning-back process

Dongxu Ma[1], Ming Ji[2], Hongbo Yi[2], Qingyu Wang[1], Fu Fan[1], Bo Feng[1, 3], Mengjie Zheng[4], Yiqin Chen[1, 3, *], Huigao Duan[1, 3, *]

[1] College of Mechanical and Vehicle Engineering, Hunan University, Changsha 410082, Hunan Province, China

[2] IBD Technology Co., Ltd., Zhongshan, 528437, Guangdong Province, China

[3] Greater Bay Area Institute for Innovation, Hunan University, Guangzhou 511300, Guangdong Province, China

[4] Jihua Laboratory, Foshan 528000, Guangdong Province, China

*Corresponding author: chenyiqin@hnu.edu.cn; duanhg@hnu.edu.cn

1. Topography evolution of Ag film during IBS deposition without and with TiO_x seed layer.

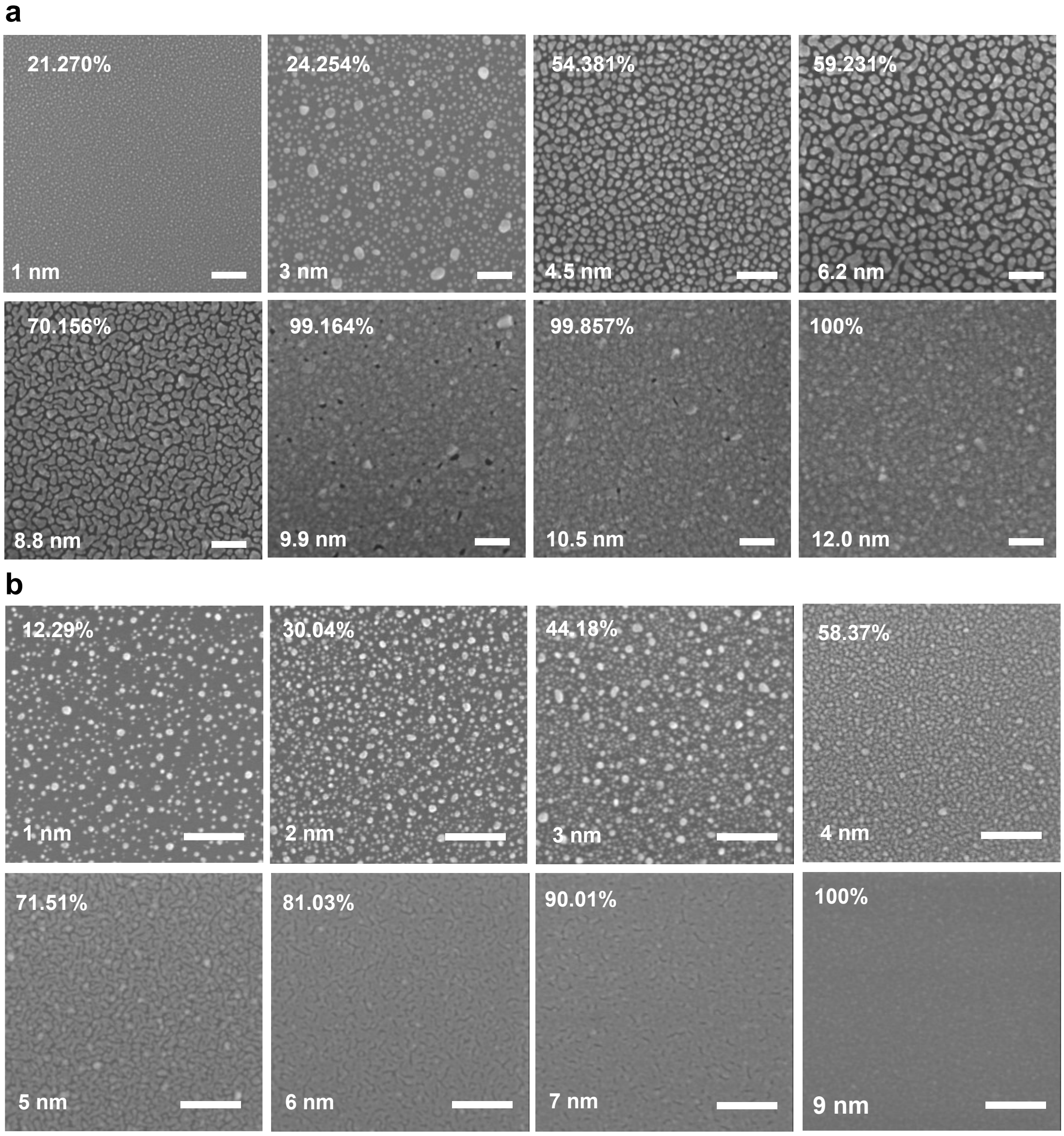

Supplementary Figure 1 | Topography evolution of Ag film growth without and with TiO_x seed layer using IBS deposition. Electron micrograph galleries for demonstrating the growth process of unseeded (a) and seeded Ag film (b), respectively. The pristine substrate is silicon die sliced by a single-crystalline wafer. Here, a 2-nm thick TiO_x seed layer is coated by the ALD technique before Ag deposition. The coverage calculation is based on extracted SEM images after black/white binary processing using ImageJ software. The thickness is determined by the extrapolation of the sputtering rate. Scale bars: 200 nm.

2. Further reducing the thickness of the UTAF beyond 4.5 nm.

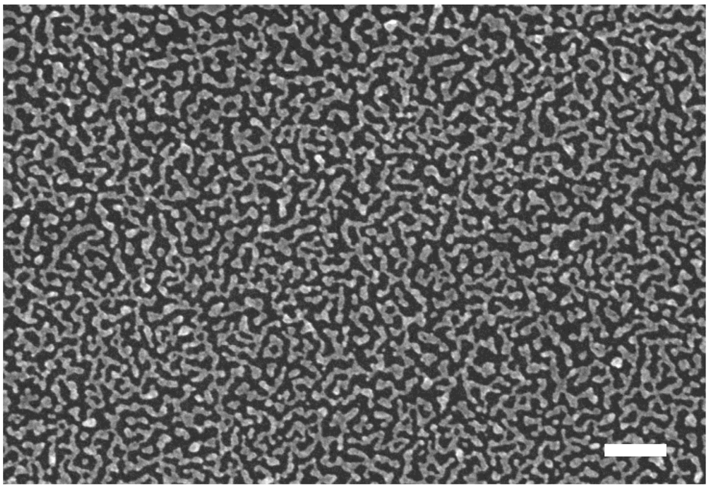

Supplementary Figure 2 | Morphology of 3-nm-thick UTAF. The result is obtained by further thinning the 4.5-nm-thick UTAF sample. Scale bar: 200 nm.

3. Change of surface roughness for IBS-deposited Ag film during thinning back.

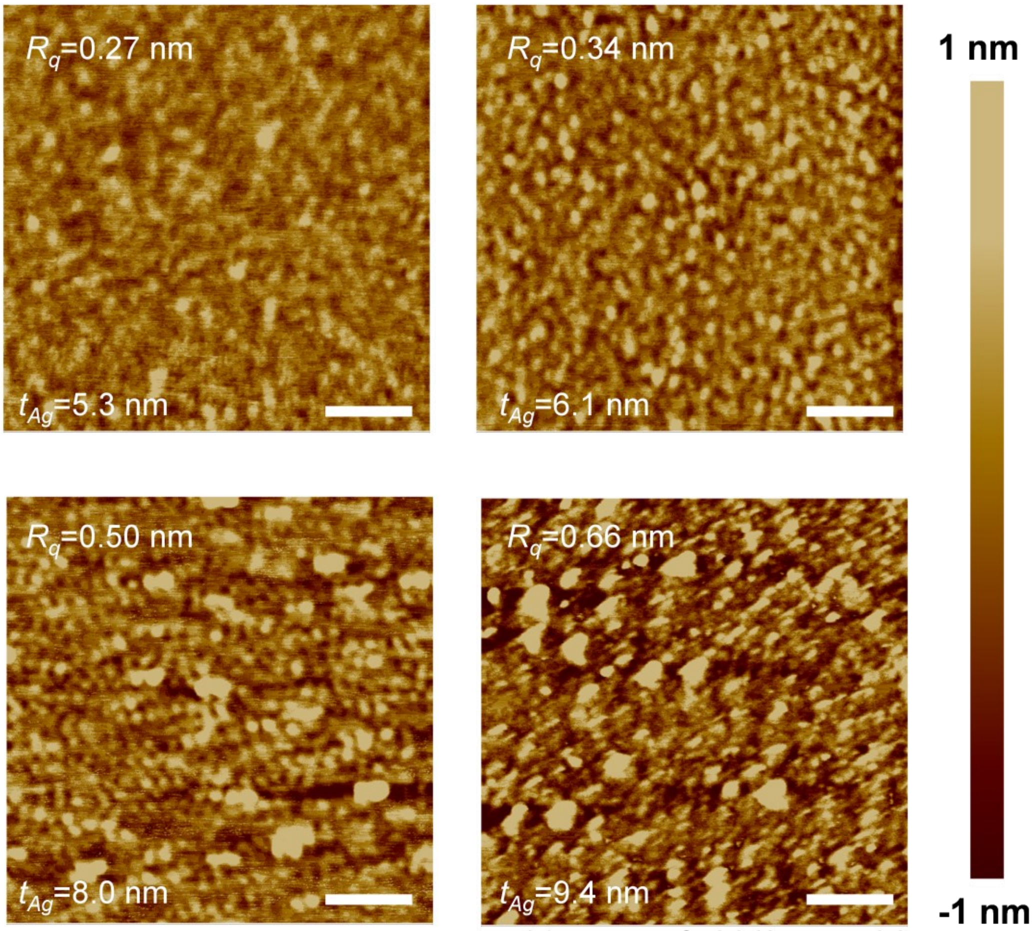

Supplementary Figure 3 | AFM topography mappings of Ag film during thinning-back procedure. All AFM mappings are performed with the resolution of 512 scanning lines on the vertical axis and 512 pixels on each lateral line. Scanning area for each sample is fixed to be 1 µm × 1 µm. The labelled values in thickness are the optical thickness which is determined by ellipsometry measurements. Scale bars: 200 nm.

4. Morphology of the thinned-back UTAFs based on the deposited Ag film by magnetron sputtering and thermal evaporation, respectively.

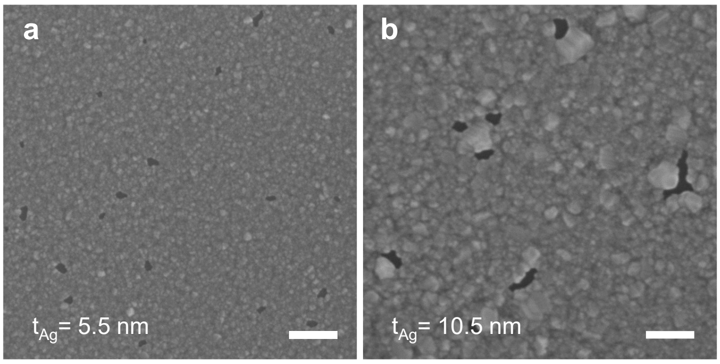

Supplementary Figure 4 | SEM images of thinned-back UTAFs based on the deposited continuous Ag films by magnetron sputtering (a) and thermal evaporation (b), respectively. The same thickness of deposited Ag films is set to be 15 nm, and the thinned-back thickness is a nominal value based on the etching rate of ion-beam polishing. Scale bars: 200 nm.

5. Morphology of 4.5-nm thick Ag film fabricated by direct IBS deposition on 2-nm TiO_x seed layer.

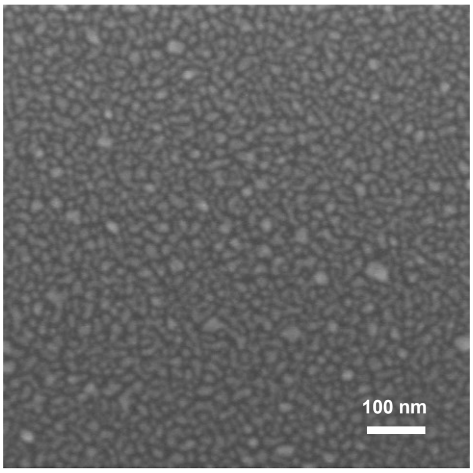

Supplementary Figure 5 | Morphology of seeded Ag film at the thickness t_{Ag}=4.5 nm, which is directly deposited by IBS. The seed layer is ALD-coated TiO_x.

Supplementary Table 1. Average visible transmittance of UTAFs varying the reduced thickness by ion-beam thinning.

Ag thickness (nm)	T_{avg} (%)
4.5	82
6.5	80
8.3	75
9.3	73
10.5	70
12.5	62

$$T_{avg} = \frac{\int_{400}^{800} T(\lambda)\, d\lambda}{800 - 400}$$

6. X-ray diffraction analysis for reduced Ag film with different thicknesses using ion beam thinning-back process.

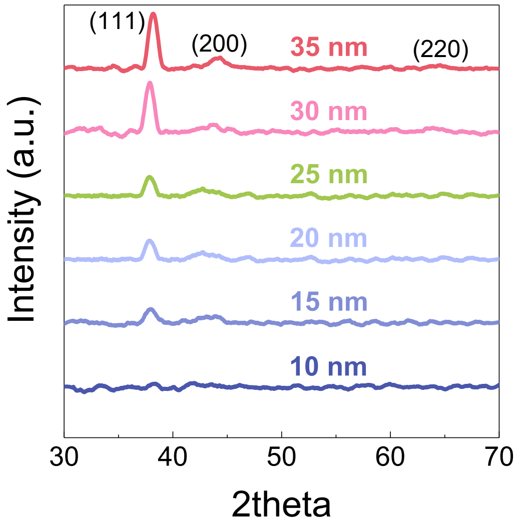

Supplementary Figure 6 | Ex-situ XRD characterization of thinned Ag film with different residue thicknesses. The thickness of prefabricated Ag film is deposited by the IBS technique. These thicknesses are determined by ellipsometry characterization.

7. Stability of the 4.5-nm UTAF when taken out of the IBS chamber.

Supplementary Table 2. The average visible transmittance and sheet resistance of 4.5-nm-thick UTAF when taken out of the IBS chamber in a week.

**Day**	**Average visible transmittance (%)**	**Sheet resistance (Ω sq^{-1})**
1st day	83.80	18.35±0.68
2nd day	83.76	18.81±0.76
6th day	83.62	19.38±1.01
7th day	83.61	19.87±0.90

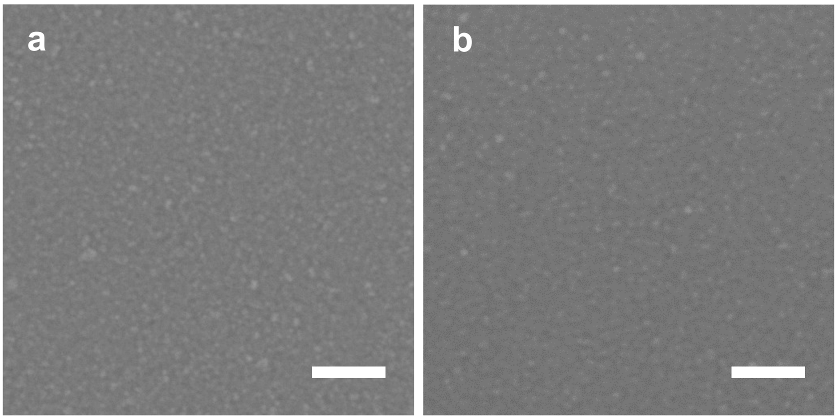

Supplementary Figure 7 | The surface morphology of as-fabricated (a) 4.5-nm UTAF and that after 7 days (b) when taken out of the IBS chamber and placed in air atmosphere (~16°C, 40% relative humidity). Scale bars: 200 nm.

8. Morphology of commercial ITO and 9-nm-thick UTAF after static folding test.

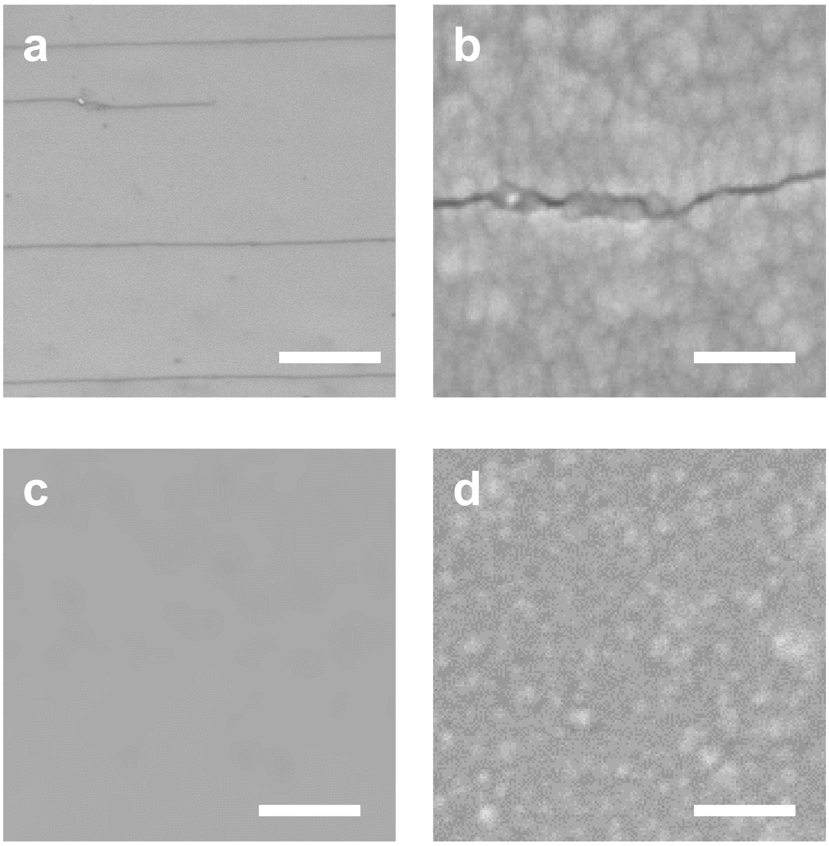

Supplementary Figure 8 | Topography characterization of commercial ITO and 9-nm-thick UTAF after static folding test with 3.5-mm curved radius using SEM and optical microscopy. **a, b** Photograph and electron micrograph of ITO. **c, d** Corresponding topographic images of UTAF. Scale bars: 25 μm in panels **a** and **c**; 100 nm in panels **b** and **d**.

9. The antireflection result of UTAF based on the ZnO/Ag/ZnO (ZAZ) structure.

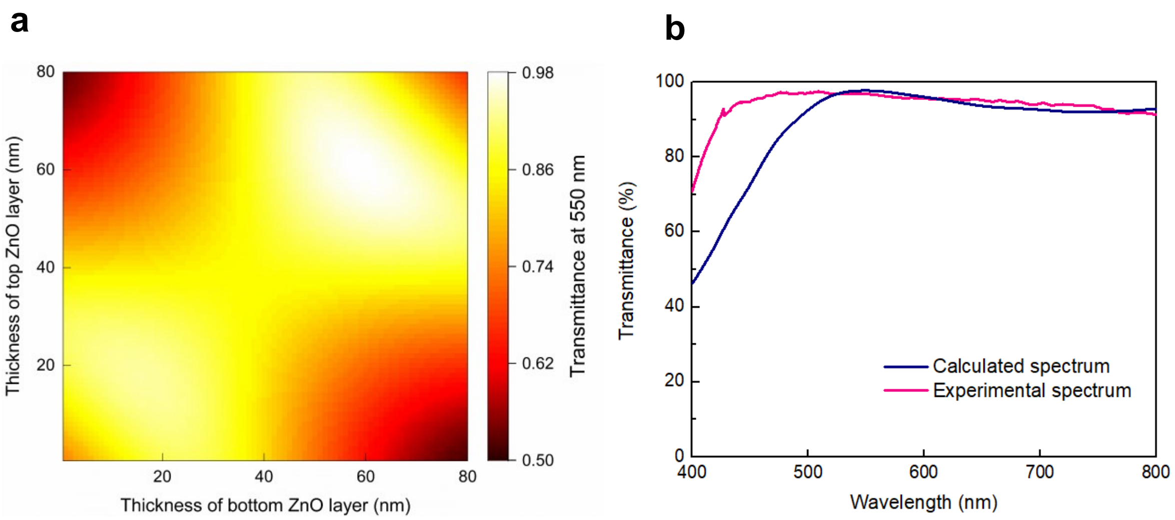

Supplementary Figure 9 | The design of antireflection for UTAF based on ZAZ structure. (a) Calculated T_{550} mapping of ZAZ as a function of top and bottom t_{ZnO} variables. Ag thickness t_{Ag} is fixed to be 8 nm. (b) The calculated spectrum and experimental spectra of ZAZ structure with setting thicknesses of 61 nm top t_{ZnO}/8 nm t_{Ag}/59 nm bottom t_{ZnO}.

Supplementary Table 3. The performance of ZAZ compared with other electrodes.

**Techniques**	$T_{550\,nm}$ **(%)**	R_s **(Ω sq^{-1})**	***FoM***	**Reference**
ITO	85.7	13.36	176	[1]
AgNWs	92	32	138	[2]
Graphene	97	125	98	[3]
CNT	74	22	53	[4]
Ag grid	90	75	46	[4]
ZnO/Ag/ZnO (61/8/59 nm)	96.8	10.16	1132	Our work

Note: FoM=188.5/(R_s×($T_{550}^{-1/2}$-1)

10. Morphology of 7-nm thick Au film fabricated by ion beam thinning-back process.

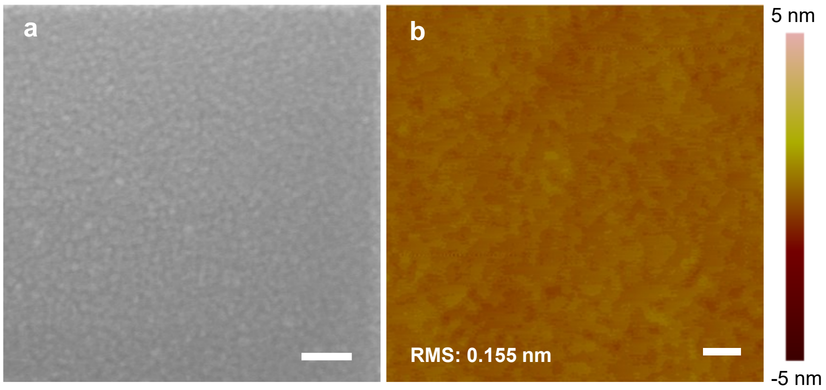

Supplementary Figure 10 | Fabricated 7-nm-thick Au film by ion beam thinning-back process. **a** SEM morphology and **b** AFM topography of ultrathin Au film on a single-polishing silicon substrate. The surface roughness is 0.155 nm RMS in statistics. The scanning resolution in AFM topography mapping is 512×512 pixels in 1×1 μm². The thickness is the optical equivalent value which is determined by spectroscopic ellipsometry. Scale bars: 100 nm.

11. Diagram of EDS line-scan mapping for Ag and Ti normal to its interface.

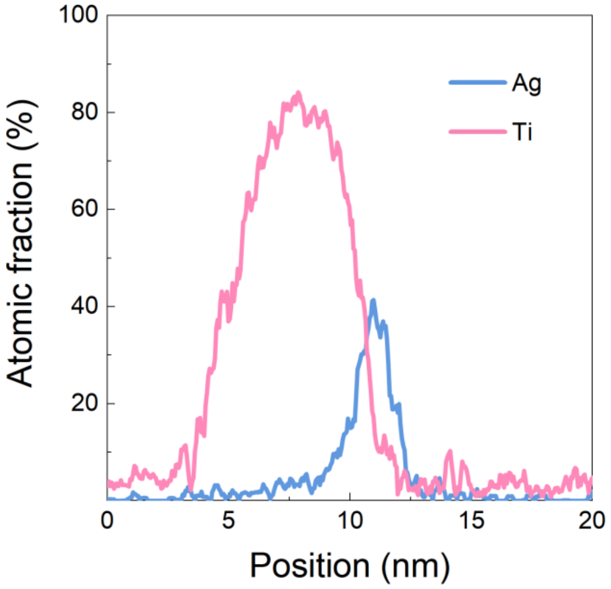

Supplementary Figure 11 | The Ag and Ti distribution analysis on the normal line of UTAF on the TiO_x wetting layer. The line-scan mapping is performed in XTEM.

Supplementary References

1. Lim, J. W. et al. Effect of geometric lattice design on optical/electrical properties of transparent silver grid for organic solar cells. *Opt. Express* **22**, 26891-26899 (2014).
2. Ge, Y. et al. Direct room temperature welding and chemical protection of silver nanowire thin films for high performance transparent conductors. *J. Am. Chem. Soc.* **140**, 193-199 (2018).
3. Bae, S. et al. Roll-to-roll production of 30-inch graphene films for transparent electrodes. *Nat. Nanotechnol.* **5**, 574-578 (2010).
4. Guo, C. F. & Ren, Z. Flexible transparent conductors based on metal nanowire networks. *Mater. Today* **18**, 143-154 (2015).
